# Topotecan for Relapsed Small-cell Lung Cancer: Systematic Review and Meta-Analysis of 1347 Patients

**DOI:** 10.1038/srep15437

**Published:** 2015-10-21

**Authors:** Nobuyuki Horita, Masaki Yamamoto, Takashi Sato, Toshinori Tsukahara, Hideyuki Nagakura, Ken Tashiro, Yuji Shibata, Hiroki Watanabe, Kenjiro Nagai, Miyo Inoue, Kentaro Nakashima, Ryota Ushio, Masaharu Shinkai, Makoto Kudo, Takeshi Kaneko

**Affiliations:** 1 Department of Pulmonology, Yokohama City University Graduate School of Medicine, Yokohama, Japan; 2 Respiratory Disease Center, Yokohama City University Medical Center, Yokohama, Japan

## Abstract

Topotecan is the most reliable chemotherapy regimen for relapsed small-cell lung carcinoma (SCLC). The efficacy and adverse effects of topotecan as reported by previous studies varied greatly. The inclusion criterion was a prospective study that was able to provide data for 6-month over-all survival (OS) rate, 1-year OS rate, objective responses, and/or adverse effects of single agent topotecan as a second line chemotherapy for SCLC, written in English language as a full article. Any topotecan regimen were allowed. Binary data were meta-analyzed with the random-model generic inverse variance method. We included 14 articles consisted of 1347 patients. Pooled values were estimated as follows. <Refractory relapse> Six-month OS rate: 37% (95% CI: 28–46%). One-year OS rate: 9% (95% CI: 5–13%). Response rate: 5% (95% CI: 1–8%). <Sensitive relapse> Six-month OS rate: 57% (95% CI: 50–64%). One-year OS rate: 27% (95% CI: 22–32%). Response rate: 17% (95% CI: 11–23%). <Adverse effect> Grade III/IV neutropenia 69% (95% CI: 58–80%). Grade III/IV thrombopenia 41% (95% CI: 34–48%). Grade III/IV anemia 24% (95% CI: 17–30%). Non-hematorogical events were rare. Chemotherapy-related death 2% (95% CI: 1–3%). In conclusion, Topotecan provided a possibly promising outcome for sensitive-relapse SCLC and poor outcome for refractory relapse SCLC. Adverse events were mainly hematological.

Lung cancer is the leading cause of cancer-related mortality in the world^1^. Patients with small-cell lung cancer (SCLC) almost always have a history of smoking and are generally very chemotherapy sensitive. Good sensitivity to chemotherapy and radiotherapy are features of SCLC. However, most patients who have initially responded to chemotherapy and radiotherapy eventually experience recurrence of the cancer in a few months^2^,^3^,^4^,^5^. Traditionally, the relapse of SCLC from the first line chemotherapy has been divided into two categories: refractory relapse, which occurs within a 60–90-day treatment-free interval (TFI) after the first line chemotherapy, and sensitive relapse, which occurs after at least 60–90 days of TFI^6^. Although the best management of recurrent SCLC is far from clear, most physicians feel that topoisomerase I inhibitor topotecan (TOP), which is sometimes referred to as nogitecan, is the most reliable chemotherapy regimens at least for sensitive relapse, because these medications have been supported by numerous clinical trials^7^,^8^,^9^,^10^,^11^,^12^,^13^,^14^,^15^,^16^,^17^,^18^,^19^,^20^. At present, TOP is the only anti-cancer drug whose efficacy for relapsed SCLC has been proved in a randomized controlled trial (RCT) that used the best supportive care arm as a comparator^13^. Some RCTs compared the efficacy of TOP for relapsed SCLC and that of other regimens, namely amurubicin^11^,^12^,^17^. However, no medication has been clearly demonstrated to be superior to TOP.

The efficacy and adverse effects (AE) of TOP are of considerable interest for all physicians who take care of patients with SCLC. Nonetheless, the efficacy and AE of TOP as reported by previous studies have seemed to vary greatly. Therefore, we tried to perform a systematic review and a meta-analysis to provide data about survival, objective response, and AEs of TOP when prescribed as the second-line chemotherapy for patients with SCLC.

## Methods

Institutional review board approval and patient consent were not required because of the review nature of this study.

### Study search

Systematic searching was conducted to find eligible articles. The inclusion criterion for a study to be included in the current meta-analysis was a prospective study that was able to provide data for the 6-month over-all survival (OS) rate, the 1-year OS rate, objective responses, and/or AEs of single agent TOP as second line chemotherapy for SCLC, written in English language as a full article. Any TOP regimen prescribed for both intravenous and oral administration was allowed. Conference abstracts and duplicate use of the same data were excluded. Two investigators independently searched for eligible articles using the PubMed, Web of Science, and Cochrane databases as of February, 2015. The following search formula was used for PubMed: (“small-cell lung cancer” OR “small-cell lung carcinoma” OR “SCLC”) AND (relapsed OR second-line OR 2nd-line OR “second line” OR “previously treated”) AND (nogitecan OR hycamtin OR topotecan OR NGT).

### Outcome

Survival was evaluated as 6-month OS rate and 1-year OS rate. If 6-month and/or 1-year survival rate was not directly provided in the article, it was estimated from the survival curve using Parmar’s method^21^.

For response analysis, response rate (RR), disease control ratio (DCR), complete response (CR), partial response (PR), stable disease (SD), progressive disease (PD), not assessable (NA) were evaluated. Both SD and no change were merged as SD. NA, non-evaluable, and early death before response assessment were counted as NA. RR included CR and PR. DCR included CR, PR, and SD. For response assessment, the total number of patients evaluated was equal to the numbers of patients with CR, PR, SD, PD, and NA.

Grade III and IV hematological toxicity including neutropenia, thrombopenia, and anemia; and grade III and IV non-hematological toxicity including fatigue, asthenia, nausea/vomiting, diarrhea, anorexia, dyspnea, and fever were assessed for AE assessment. Febrile neutropenia of any grade was counted. Death that was clearly documented as due to AE was counted. Other minor non-hematological AEs were sometimes mentioned in articles; however they were not assessed for the current systematic review. This was because very rare AEs were usually not mentioned in most articles, thus, including these very rare AEs might have resulted in publication bias. AEs were analyzed based on the number of patients, not on the number of chemotherapy courses.

Survival analysis and response analysis were conducted for patients with sensitive relapse and refractory relapse separately^6^. AE analysis was conducted for both relapses collectively.

### Statistics

Binary data were meta-analyzed with the random-model generic inverse variance method after the standard error was estimated using the Wilson score interval^22^,^23^,^24^. The heterogeneity evaluated with the I^2^ statistics was interpreted as follows: I^2^ = 0% indicates no heterogeneity, 0% < I^2^ < 25% indicates the least heterogeneity, 25% ≤ I^2^ < 50% indicates mild heterogeneity, 50% ≤ I^2^ < 75% indicates moderate heterogeneity, and 75% ≤ I^2^ indicates strong heterogeneity^25^.

For a subgroup analysis, we divided TOP regimens into three groups: intravenous days 1–5 administration, intravenous weekly administration, and oral administration. Subgroup analyses were not performed for outcomes for refractory relapse because these analyses did not include sufficient numbers of original cohorts.

All analysis was performed in Review Manager ver 5.3 (Cochrane Collaboration, Oxford, UK). Figures illustrated using Review Manager were adjusted as necessary.

## Results

### Study search

**Figure 1:**
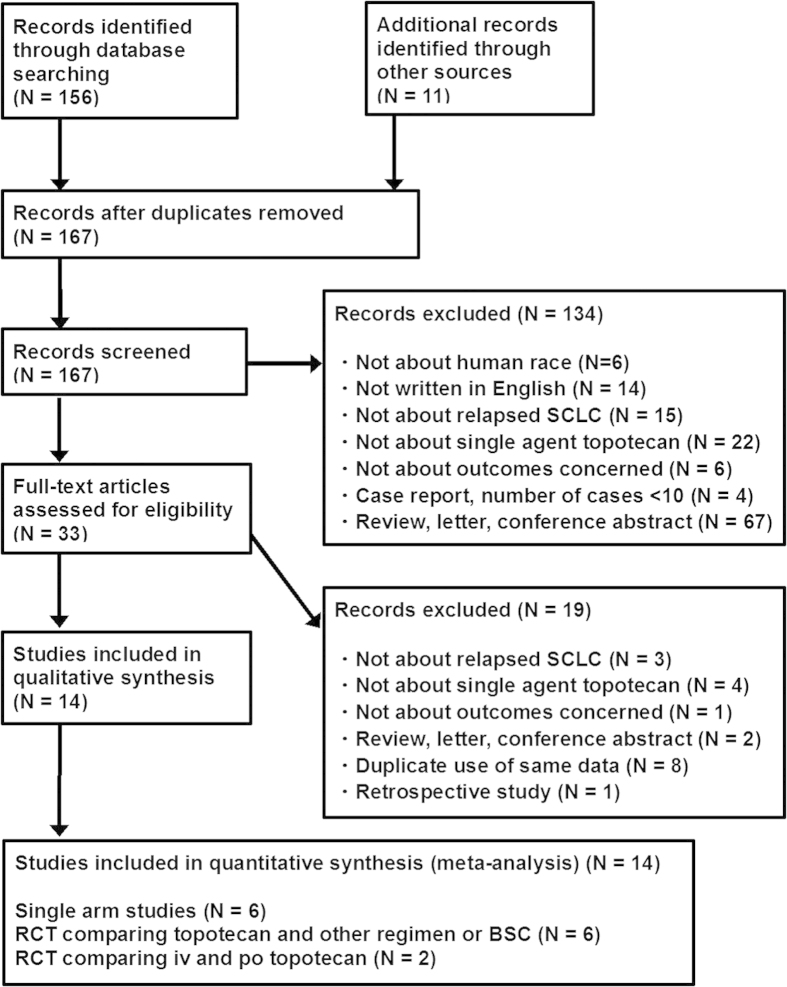
Flow chart for study search (PRISMA diagram).

Of 167 articles that met the preliminary criteria, 134 and 19 were excluded through title/abstract screening and full article scrutinizing, respectively. We found 14 eligible articles, which included six single arm studies, two RCTs that compared intravenous and oral TOP, and six RCTs that compared TOP and other regimens (Fig. 1, Table 1)^7^,^8^,^9^,^10^,^11^,^12^,^13^,^14^,^15^,^16^,^17^,^18^,^19^,^20^.

The number of patients in a study who were treated with TOP ranged from 17 to 309. The total number of patients in all the studies was 1347. In most of the studies, patients with performance status of 1 and men were the majority. Mean or median age presented for each study ranged from 58 to 68 years. Two studies did not mention types of relapse. The cutoff between refractory and sensitive relapse was 90 days except for one study that used 60 days. Intravenous TOP 1.5 mg/m^2^ on days 1–5 every 3 weeks was the most preferred regimen. Two studies from Japan used intravenous TOP in a low dose of 1.0 mg/m^2^ on day 1–5 every 3 weeks. Three studies used weekly intravenous TOP in higher doses of 4.0 or 6.0 mg/m^2^. Three studies used oral TOP 2.3 mg/m^2^ on days 1–5 every 3 weeks (Table 1).

### Refractory relapse

**Figure 2:**
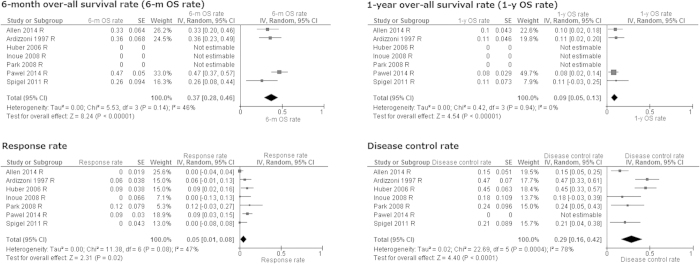
Meta-analysis for refractory relapse. SE: standard error. IV: inverse variance method. 95% CI: 95% confidence interval.

Pooled 6-month and 1-year OS rates estimated from four cohorts were 37% (95% CI 28–46%. I^2^ = 46%, *p* for heterogeneity = 0.14) and 9% (95% CI 5–13%. I^2^ = 0%, *p* for heterogeneity = 0.94), respectively (Fig. 2).

Concerning objective response, random-model meta-analysis using the generic inverse variance method suggested the following pooled values: RR 5% (95% CI 1–8%. I^2^ = 47%, *p* for heterogeneity = 0.08), DCR 29% (95% CI 16–42%. I^2^ = 78%, *p* for heterogeneity < 0.001), CR 0% (95% CI 0–2%. I^2^ = 0%, *p* for heterogeneity = 0.98), PR 3% (95% CI 0–7%. I^2^ = 40%, *p* for heterogeneity = 0.16), SD 25% (95% CI 14–36%. I^2^ = 73%, P for heterogeneity = 0.005%), PD 59% (95% CI 47–70%. I^2^ = 65%, *p* for heterogeneity = 0.02), NA 9% (95% CI 0–17%. I^2^ = 80%, *p* for heterogeneity < 0.001) (Fig. 2).

### Sensitive relapse

**Figure 3:**
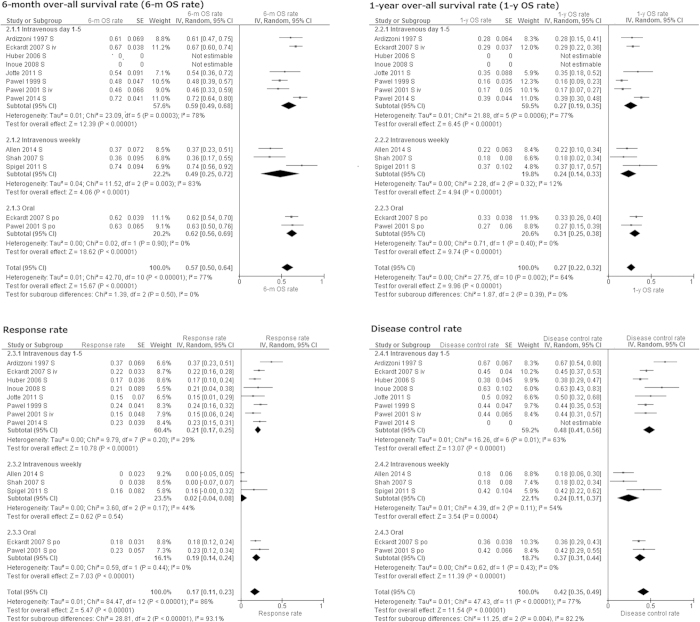
Meta-analysis for sensitive relapse. SE: standard error. IV: inverse variance method. 95% CI: 95% confidence interval.

The pooled 6-month and 1-year OS rates estimated from 11 cohorts were 57% (95% CI 50–64%. I^2^ = 77%, *p* for heterogeneity < 0.001) and 27% (95% CI 22–32%. I^2^ = 64%, *p* for heterogeneity = 0.002), respectively (Fig. 3).

Random-model meta-analysis using the generic inverse variance method suggested the following pooled values for sensitive relapse: RR 17% (95% CI 11–23%. I^2^ = 86%, *p* for heterogeneity < 0.001), DCR 42% (95% CI 35–49%. I^2^ = 77%, *p* for heterogeneity < 0.001), CR 1% (95% CI 0–1%. I^2^ = 1%, *p* for heterogeneity = 0.43), PR 14% (95% CI 8–21%. I^2^ = 85%, *p* for heterogeneity < 0.001), SD 22% (95% CI 19–24%. I^2^ = 0%, *p* for heterogeneity = 0.57), PD 48% (95% CI 40–56%. I^2^ = 82%, *p* for heterogeneity < 0.001), NA 11% (95% CI 6–16%. I^2^ = 85%, *p* for heterogeneity < 0.001) (Fig. 3).

We performed subgroup analysis dividing TOP regimen into three groups. RR of 2% (95% CI: 0–8%), and DCR of 24% (95% CI 11–37%) by intravenous weekly administration were poorer than those by other regimens with significant subgroup differences. Test for subgroup differences were following: RR, I^2^ = 93.1%, P < 0.001; DCR, I^2^ = 82.2%, P < 0.001 (Fig. 3). Nonetheless, 6-month OS rate (I^2^ = 0%, *p* for subgroup heterogeneity = 0.50), and 1-year OS rate (I^2^ = 0%, *p* for subgroup heterogeneity = 0.39) were not largely different among three TOP regimens (Fig. 3).

### Adverse effects

**Figure 4:**
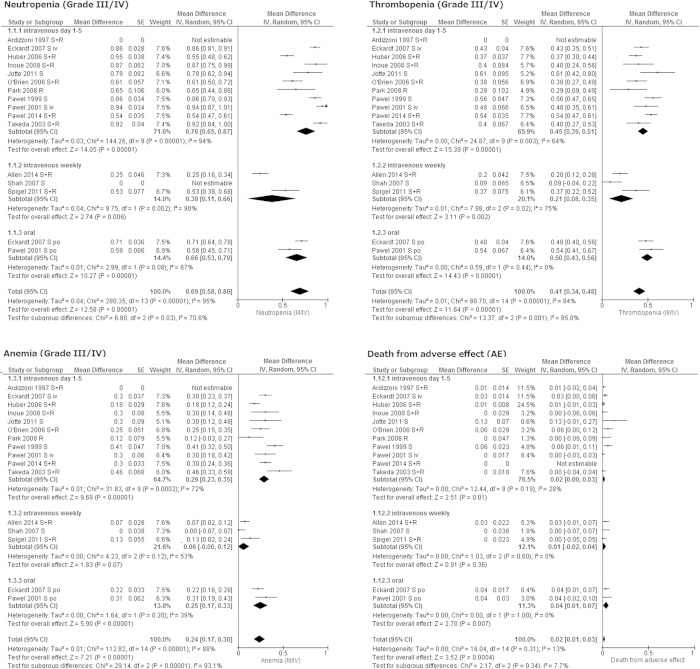
Meta-analysis for adverse effects. SE: standard error. IV: inverse variance method. 95% CI: 95% confidence interval.

Throughout the included articles, hematological AEs were more commonly observed than non-hematological AEs. The pooled grade III/IV incidence of neutropenia, thrombopenia, and anemia were 69% (95% CI 58–80%. I^2^ = 95%, *p* for heterogeneity < 0.001), 41% (95% CI 34–48%. I^2^ = 84%, *p* for heterogeneity < 0.001), and 24% (95% CI 17–30%. I^2^ = 88%, *p* for heterogeneity < 0.001), respectively. Febrile neutropenia was observed with a pooled incidence of 4% (95% CI 2–5%. I^2^ = 0%, *p* for heterogeneity = 0.88) (Fig. 4).

Subgroup analysis suggested that weekly regimen had lower frequency for hematological AEs with substantial subgroup heterogeneity. When weekly regimen was selected, pooled incidences were 38% (95% CI 11–66%) for Grade III/IV neutropenia, 21% (95% CI 8–35%) for Grade III/IV thrombopenia, and 6% (95% CI 0–12%) for Grade III/IV anemia (Fig. 4).

The pooled incidences of non-hematological AEs were as follows: fatigue 6% (95% CI 3–9%. I^2^ = 76%, *p* for heterogeneity < 0.001), asthenia 3% (95% CI 0–6%. I^2^ = 66%, *p* for heterogeneity = 0.02), nausea/vomiting 2% (95% CI 1–3%. I^2^ = 0%, *p* for heterogeneity = 0.66), diarrhea 2% (95% CI 0–4%. I^2^ = 33%, *p* for heterogeneity = 0.14), anorexia 3% (95% CI 1–5%. I^2^ = 40%, *p* for heterogeneity = 0.14), dyspnea 5% (95% CI 2–8%. I^2^ = 60%, *p* for heterogeneity = 0.01), fever 2% (95% CI 1–4%. I^2^ = 33%, *p* for heterogeneity = 0.16) (Fig. 4).

Chemotherapy-related death was observed with a pooled incidence of 2% (95% CI 1–3%. I^2^ = 13%, *p* for heterogeneity = 0.31) (Fig. 4).

## Discussion

TOP for patients with sensitive relapse SCLC could provide RR for 17% of cases, and disease control for 42% of cases, which resulted in 57% of 6-month OS rate and 27% of 1-year OS rate (Fig. 3). However, this regimen had poorer outcomes for patients with refractory relapse SCLC (Fig. 2). Hematologic AEs, especially grade III/IV neutropenia were more commonly observed than non-hematological AEs. Approximately 2% of patients died from chemotherapy (Fig. 4).

In addition to the most commonly used regimen of intravenous 1.5 mg/m^2^ on day 1–5 every three weeks, high dose weekly intravenous TOP regimens and oral TOP 2.3 mg/m^2^ on day 1–5 every three weeks have been often selected. RCTs by Eckardt *et al.* in 2007 and by Pawel *et al.* in 2001 suggested that oral and intravenous TOP on day1–5 every three weeks have similar efficacy and AE profiles^9^,^16^. Thus, both regimens could be appropriate choices of treatment for sensitive relapse SCLC (Fig. 3). Oral TOP could provide a convenient administration route with a similar AE profile to intravenous TOP (Fig. 4). Weekly intravenous TOP has been found to be a treatment with good tolerability^7^,^18^,^19^. In our analysis, frequency of hematological AEs by weekly regimen were less than that by other regimens (Fig. 4). Nonetheless, RR of 2% and DCR of 24% for sensitive relapse by intravenous weekly administration may be disappointing (Fig. 3)^18^,^19^. Considering 6-month and 1-year OS rates by weekly regimen were not inferior to those by intravenous day 1–5 regimen and oral regimens, weekly regimen could be a choice for patients who have high risk for myelosuppression.

There is still little evidence to guide the third-line chemotherapy. Nonetheless, to improve OS time, a physician provide the third-line chemotherapy for a part of patients whose disease relapsed after the second-line chemotherapy^26^,^27^. Here, a clinician faces a challenge how to select a SCLC case who will gain benefit from further treatment. Retrospective chart reviews suggested that patients with normal or low lactase dehydrogenase level, good response to second-line chemotherapy, high body mass index, higher level of hemoglobin, long time to progression after the second-line chemotherapy indicated better response to the third-line chemotherapy^26^,^27^.

In 2014, two reports that were potentially conflicting with each other were published. Lara *et al.* analyzed 329 patients with extensive-stage SCLC who progressed after platinum-based chemotherapy^28^. This study indicated that platinum-sensitivity status may no longer be strongly associated with progression-free survival and OS. On the other hand, Ardizzoni *et al.* reported a retrospective study with 631 relapsed SCLC cases^6^. This concluded that the separation of relapsed SCLC into two types of relapses based on a TFI cut-off of 60 days was valid and could be a standard of care. The current analysis, though mostly using a TFI cut-off of 90 days, also demonstrated that all of 6-month OS rate, 1-year OS rate, RR and DCR were more favorable for patients with sensitive relapse than those with refractory relapse.

Few limitations of the current study should be mentioned. First, the meta-analysis was performed as aggregated data meta-analysis. If we could have obtained individual data from all the original studies, individual patient data meta-analysis would have been preferred. Second, regimen comparison was not done by head-to-head manner. Third, strong heterogeneities for some outcomes made it difficult to interpret results. However, we believe the current meta-analysis provided useful information for clinicians.

In conclusion, TOP provided a possibly promising outcome for patients with sensitive-relapse SCLC and poor outcome for patients with a refractory relapse SCLC. Adverse events were mainly hematological. We believe these data will be informative for physicians who take care of patients with relapsed SCLC.

## Additional Information

**How to cite this article**: Horita, N. *et al.* Topotecan for Relapsed Small-cell Lung Cancer: Systematic Review and Meta-Analysis of 1347 Patients. *Sci. Rep.* **5**, 15437; doi: 10.1038/srep15437 (2015).

## Figures and Tables

**Table 1:** Summary of included studies.

**Study**	**Design**	**Sub-group**	**N**	**PS(%) 0/1/2**	**Age**	**men%**	**TOP regimen**
Allen 2014^7^	RCT, iv-TOP vs iv-TOP+ZA, P2	S	41	41/59/0	60	32%	iv 4 mg/m^2^, d 1/8/15 every 3 w
		R	51	37/63/0	64	67%	iv 4 mg/m^2^, d 1/8/15 every 3 w
Ardizzoni 1997^8^	Single arm, iv-TOP	S	46	33/52/15	58	74%	iv 1.5 mg/m^2^, d 1–5 every 3 w
		R	47	17/62/21	58	64%	iv 1.5 mg/m^2^, d 1–5 every 3 w
Eckardt 2007^9^	RCT, oral-TOP vs iv-TOP, P3	oral	155	31/56/13	63	64%	oral 2.3 mg/m^2^, d 1–5 every 3 w
		iv	154	23/65/12	62	64%	iv 1.5 mg/m^2^, d 1–5 every 3 w
Huber 2006^10^	Single arm, iv-TOP		170	21/56/23	61	78%	iv 1.25 mg/m^2^, d 1–5 every 3 w
Inoue 2008^11^	RCT, iv-TOP vs AMR, P2		30	57/30/13	64	83%	iv 1.0 mg/m^2^, d 1–5 every 3 w
Jotte 2011^12^	RCT, iv-TOP vs AMR, P2		26	39/54/8	68	42%	iv 1.5 mg/m^2^, d 1–5 every 3 w
O’Brien 2006^13^	RCT, oral-TOP vs BSC, P3		71	11/62/27	60	73%	oral 2.3 mg/m^2^, d 1–5 every 3 w
Park 2008^14^	Single arm, iv-TOP		17	6/77/18	68	94%	iv 1.5 mg/m^2^, d 1–5 every 3 w
Pawel 1999^15^	RCT, iv-TOP vs CAV		107	17/60/23	NA	57%	iv 1.5 mg/m^2^, d 1–5 every 3 w
Pawel 2001^16^	RCT, oral-TOP vs iv-TOP, P2	oral	52	19/65/15	60	75%	oral 2.3 mg/m^2^, d 1–5 every 3 w
		iv	54	33/39/28	58	80%	iv 1.5 mg/m^2^, d 1–5 every 3 w
Pawel 2014^17^	RCT, iv-TOP vs AMR, P3		213	34/64/2	61	60%	iv 1.5 mg/m^2^, d 1–5 every 3 w
Shah 2007^18^	Single arm, iv-TOP, P2		22	18/73/9	63	55%	iv 4 mg/m^2^, d 1/8/15 every 4 w
Spigel 2011^19^	Single arm, iv-TOP, P2		38	26/74/0	64	53%	iv 6 mg/m^2^, d 1/8/15/22/29/36 every 8 w
Takeda 2003^20^	Single arm, iv-TOP, P2		53	22/60/18	64	68%	iv 1.0 mg/m^2^, d 1–5 every 3 w

S: sensitive relapse. R: refractory relapse. RCT: randomized controlled trial. TOP: topotecan. iv: intravenous. ZA: ziv-aflibercept. AMR: amurubicin. BSC: best supportive care. P2/3: phase 2/3. N: number of patients. PS: performance status. Age: mean or median age was presented. d: day. w: week.
